# “Validation of a cyberbullying questionnaire as a screening tool for other forms of intimate-partner violence towards young women”

**DOI:** 10.1186/s12889-021-11646-3

**Published:** 2021-09-08

**Authors:** Lucía Hernández García, Myrian Pichiule Castañeda, Luisa Lasheras Lozano, Marisa Pires Alcaide, María Ordobás Gavín, Ana Gandarillas Grande

**Affiliations:** 1 Epidemiology Area, General Subdirectorate of Epidemiology, General Directorate of Public Health, Ministry of Health, Community of Madrid, Madrid, Spain; 2 Preventive Medicine Unit, University Hospital 12 de Octubre, Community of Madrid, Madrid, Spain; 3 Health Promotion Unit, General Subdirectorate of Health Promotion, Prevention and Education for Health, General Directorate of Public Health, Ministry Education for Health, Community of Madrid, Madrid, Spain

**Keywords:** Cyber-dating abuse, Gender-based violence, Intimate-partner violence, Health surveys, Validation study, Mass screening, Young adult

## Abstract

**Background:**

The use of electronic media is widespread among young people and is a potential tool for the perpetration of intimate-partner violence (IPV) towards women. The aim of this study is to validate two questions focused on harassment and control by electronic tools (HCE-2) as a screening tool for the detection of IPV in young women.

**Methods:**

The data source was the third Community of Madrid IPV survey in 2014. The screening tool consisted of two questions with five possible answers prepared by a group of experts. As the gold standard we used the definition of intimate partner violence based on a 26- question survey. The validity indices (with 95% confidence intervals) were compared between two age groups: 18–24 and 25–29 years.

**Results:**

Six hundred ninty-four women were sampled. The response rate was 68.7%, and 477 surveys were analyzed. The prevalence of IPV was 10.7% (95% CI: 8.2–13.8). HCE-2 was positive in 5.9% (95% CI: 4.1–8.4). The overall efficiency of the test was 93.5% (95% CI: 91.1–96.7), sensitivity 47.1% (95% CI: 33.7–60.8), specificity 99.1% (95% CI: 97.5–99.6), and positive predictive value 85.7% (95% CI: 67.1–94.6). The best validity indices of the questionnaire were observed in women aged 18 to 24 years: overall efficiency of the test 95.1% (95% CI: 92.6–97.7), sensitivity 62.5% (95% CI: 44.5–77.6), specificity 99.6% (95% CI: 97.0–99.9), and positive predictive value 95.2% (95% CI: 71.7–99.4).

**Conclusions:**

The existing need to improve the detection of IPV in young women and the good validity indices observed here justify the recommendation of the HCE-2 questionnaire as a screening tool in young women.

## Background

Gender-based violence is a social and health problem widely described in the literature and is recognized as a violation of basic human rights, with psychological and physical consequences for both the victim and their environment [1, 2]. Despite the social changes that have occurred in recent decades, this type of violence is still present in all stages of life, with intimate-partner violence (IPV) being the most common form and young women being the most frequently affected demographic group [3]. According to the national survey in Spain (10,171 women survey), 26.4% of women have suffered psychological violence from their partner or ex-partner over the course of their lives, and 9.5% have experienced it in the last year, while in women aged 16 to 24 years, these percentages are 38.3 and 21.1%, respectively [3]. In addition to suffering violence more often, young women seem to be more vulnerable to its consequences [4].

There are many ways this violence is perpetrated, classically grouped into psychological, physical, and sexual violence. In recent years, electronic media have gained prominence in interpersonal communications, especially in youths, constituting a new possible mechanism for perpetrating violence [5]. The different types of violence are not exclusive; they often coexist, and a correlation has been observed with cyberbullying in dating partners [6–8].

The identification of cases of IPV is fundamental both for surveillance and for an early and adequate approach that minimizes its consequences and facilitates access to specific resources. The Spanish Organic Law 1/2004 [9] adopts measures to optimize the early detection of gender-based violence in a healthcare setting, however the screening tools studied have poorer validity indices in the young population [4, 10], which reflects the need to adapt screening to different stages of life. The Ministry of Health considers it necessary to develop tools for measuring cyber-dating abuse as a form of IPV in young people [11], on this line, one of the measures included in the national strategy for eradicating violence against women is to study cyberbullying as a new form of gender violence among young people and young couples [12].

The objective of this study is to validate two questions on the perceptions of harassment and control by electronic tools (HCE-2) as a population screening tool for IPV in youths.

## Methods

This was a validation study of a screening questionnaire on IPV towards women based on a cross-sectional population-based study.

### Study population and data collection

The source of information was the third survey to study the magnitude, trend, and health impact of IPV towards women in the Community of Madrid, 2014 [13]. The data were collected between December 2013 and February 2014. The sampling frame was the Individual Health Card database, which allows access to the health system to almost all of the population. The sample was stratified via proportional affiliation to the strata determined by the crossing of three geographical areas, age groups (four groups) and country of birth (born in Spain or outside Spain). The information was collected through a computer-assisted telephone interview.

This study included women in the sample aged 18 to 29 who reported having had a partner or contact with a former partner of the opposite gender in the last year.

### Study variables

#### Definition of IPV

Cases of violence were defined by a questionnaire of 26 questions selected from the ENVEFF questionnaire (*Enquête national sur le violences envers les femmes en France*) for the detection of psychological and sexual violence and the CTS-1 (Conflict Tactics Scales) questionnaire for the detection of physical violence. These questionnaires were used as references in this study. The Spanish version was validated in 2004 in women in the Community of Madrid through an in-depth interview conducted by two trained psychologists. They found that this questionnaire showed a sensitivity for the detection of IPV of 80.4% (79.3–81.6) and a specificity of 90.0% (88.9–91.0) [14]. Only one relationship (the most recent) was considered per participant in the study.

#### Definition of cyberbullying

To explore the perception of harassment and control by electronic tools by the couple, the HCE-2 questionnaire was designed. It consisted of two questions developed ad hoc by a group of experts, shown in Table 1, with five possible responses on a Likert scale. Cyberbullying was considered to be present in women who scored greater than or equal to 1, with a positive response of “sometimes”, “often” or “constantly/always/systematically” to at least one of the two questions.

**Table 1:** Questionnaire on perceived harassment and control by electronic tools and scoring (HCE-2) (English version and Spanish version)

**Scoring of the harassment and control questionnaire by electronic tools**				
Question 1. Have you felt harassed/overwhelmed by text messages or emails that he sends/has sent you?				
Never	Rarely	Sometimes	Often	Constantly/always/systematically
0	0	1	1	1
Question 2. Have you felt overwhelmed because he controlled calls or messages on your cell phone or email?				
Never	Rarely	Sometimes	Often	Constantly/always/systematically
0	0	1	1	1
**Positive case: 1 or 2 points in total**				
**Cuestionario de percepción de acoso y control a través de medios electrónicos y forma de puntuación (ACE-2) (Spanish versión)**				
**Puntuación del cuestionario de acoso y control a través de medios electrónicos**				
Pregunta 1. ¿Se ha sentido acosada/ agobiada a través de mensajes de móvil o de correos electrónicos que él le envía/ enviaba?				
Nunca	Rara vez	Algunas veces	Muchas veces	Constantemente/ siempre/ sistemáticamente
0	0	1	1	1
Pregunta 2. ¿Se ha sentido agobiada debido a que le controlaba las llamadas o mensajes del móvil o su correo electrónico?				
Nunca	Rara vez	Algunas veces	Muchas veces	Constantemente/ siempre/ sistemáticamente
0	0	1	1	1
**Caso positivo: 1 o 2 puntos en total**				

#### Sociodemographic variables

Age was stratified in two groups (18–24 and 25–29 years). The country of birth of the woman and her partner was either Spain or outside of Spain. Maximum level of schooling attained, employment status, type of relationship (partner or ex-partner), living with the partner or not, and whether or not they had children were also surveyed.

### Statistical analysis

The population prevalence of IPV in the year before the survey, the prevalence of cyberbullying, and their 95% confidence intervals (95% CIs) were estimated. Several validity indices of the cyberbullying questionnaire for the detection of IPV were calculated: sensitivity, specificity, positive predictive value (PPV), negative predictive value (NPV), positive odds ratio (POR), and negative (NOR), the overall efficiency of the test (percentage of cases correctly classified), and their 95% CIs. For the comparison of qualitative variables, the chi-squared test [15]. For all tests, statistical significance was accepted at the 0.05 level. The analysis was performed with the statistical program STATA version 15 (StataCorp, College Station, Texas, USA 2017).

The survey protocol was approved by the Ethics Committee for Clinical Research at La Princesa University Hospital in Madrid. All participants gave their verbal consent to participate at the beginning of the survey.

## Results

The overall response rate of the survey was 60.5% (women 18–70 years), with the highest response rate among women 18–24 years (68.7%) [2]. The HCE-2 questionnaire was open to all women, without a filtering question, and all 477 women included in the analysis responded to it.

The sociodemographic characteristics of the young women according to age group are presented in Table 2. Significant differences were found between women aged 18–24 and 25–29 years in schooling level, employment status, and whether they had children. Among the youngest age group, most were students (34.7%), while in the group aged 25 to 29 years, the majority of women were employed (65.1%).

**Table 2:** Sociodemographic characteristics by age group

	All (***n*** = 477)		18–24 years (***n*** = 268)		25–29 years (***n*** = 209)		
Variable	Frequency	Percentage (%)	Frequency	Percentage (%)	Frequency	Percentage (%)	***p***-value
**Schooling level**							< 0.001
High	140	29.5	57	21.4	83	40.1	
Medium	276	58.2	178	66.7	98	47.3	
Low	58	12.2	32	12.0	26	12.6	
**Place of birth**							0.073
Spain	357	74.8	209	78.0	148	70.8	
Other	120	25.2	59	22.0	61	29.2	
**Employment situation**							< 0.001
Paid work	233	48.9	97	36.2	136	65.1	
Unemployed	110	23.1	66	24.6	44	21.1	
Student	102	21.4	93	34.7	9	4.3	
Housewife	32	6.7	12	4.5	20	9.6	
**Type of relationship**							0.052
Current partner	395	82.8	214	79.9	181	86.6	
Ex-partner	82	17.2	54	20.2	28	13.4	
**Living together**							< 0.001
Yes	184	38.6	52	19.4	132	63.2	
No	293	61.4	216	80.6	77	36.8	
**Children**							< 0.001
Yes	82	17.2	28	10.5	54	25.8	
No	395	82.8	240	89.6	155	74.2	
**Country of partner/ex-partner**							0.508
Spain	361	75.7	206	76.9	155	74.2	
Other	115	24.1	61	22.8	54	25.8	

Regarding intimate relationships, in most cases the relationship with the current partner (82.8%) was explored, and in the rest with a former partner. The women aged 18–24 years lived with their partners less often than older women (19.4% vs. 63.2%), and young women had children less often than older women (10.5% vs. 25.8%).

The distribution of the responses to the harassment and control questions and their prevalence are shown in Table 3. In the sample, 51 cases of IPV were detected (prevalence 10.7%). The prevalence was higher among the youngest age group (11.9%; 95% CI: 8.6–16.4) than the older (9.1%; 95% CI: 5.-13.8), but this was not statistically significant.

**Table 3:** Distribution of responses to the questionnaire on harassment and control by electronic tools

	All (***n*** = 477)			18–24 years (***n*** = 268)			25–29 years (***n*** = 209)		
	**%**	**95% CI**		**%**	**95% CI**		**%**	**95% CI**	
**Intimate-partner violence towards women**	10.7	8.2	13.8	11.9	8.6	16.4	9.1	5.9	13.8
**Harassment and/or control**	5.9	4.1	8.4	7.8	5.2	11.7	3.3	1.6	6.9
**Question 1. Harassment**									
Have you felt harassed/overwhelmed by text messages or emails that he sends/has sent you?	**%**	**95% CI**		**%**	**95% CI**		**%**	**95% CI**	
Never	94.8	92.3	96.4	92.5	88.7	95.1	97.6	94.3	99.0
Rarely	1.9	1.0	3.6	2.2	1.0	4.9	1.4	0.5	4.4
Sometimes	2.1	1.1	3.9	3.0	1.5	5.9	1.0	0.2	3.8
Often	0.4	0.1	1.7	0.7	0.2	3.0	0.0	–	
Constantly/always	0.8	0.3	2.2	1.5	0.6	3.9	0.0	–	
Never/rarely	96.6	94.6	97.9	94.8	91.3	96.9	99.0	96.2	99.8
Sometimes/often/constantly/always	3.4	2.1	5.4	5.2	3.1	8.6	1.0	0.2	3.8
**Question 2. Control**									
Have you felt overwhelmed because he controlled calls or messages on your cell phone or email?	**%**	**95% CI**		**%**	**95% CI**		**%**	**95% CI**	
Never	91.6	88.8	93.8	88.4	84.0	91.8	95.7	91.9	97.8
Rarely	2.9	1.7	4.9	4.1	2.3	7.3	1.4	0.5	4.4
Sometimes	3.6	2.2	5.7	4.1	2.3	7.3	2.9	1.3	6.3
Often	1.0	0.4	2.5	1.9	0.8	4.4	0.0	–	
Constantly/always	0.8	0.3	2.2	1.5	0.6	3.9	1.0	–	
Never/rarely	94.5	92.1	96.3	92.5	88.7	95.1	97.1	93.7	98.7
Sometimes/often/constantly/always	5.5	3.7	7.9	7.5	4.9	11.3	2.9	1.3	6.3

The HCE-2 questionnaire was positive in 5.9% of women (95% CI: 4.1–8.4), that means nearly 6% of young women perceive a situation of harassment or control by their partner through electronic tools. When examining the two questions independently, there was a higher rate of positive responses to the control question (5.5%) than to the harassment question (3.4%). Among those who reported harassment by their partner, most responded “Sometimes” (2.1%), followed by “Constantly or always” (0.8%). Women who reported being controlled by their partners also responded more frequently “Sometimes” (3.6%) followed in this case by “Often” (1.0%).

The prevalence of cyberbullying was twice as high in the younger women (18–24 years) as in women aged 25–29 years (7.8% vs. 3.3%). This difference was most notable on the harassment question, with positive response rate of 5.2% in the younger group and 1.0% in the older group.

Female victims of cyberbullying were also classified as victims of IPV in 85.7% of cases. This percentage was higher in the group aged 18–24 years (95.2%); of these, only one woman out of the 28 who scored positive for cyberbullying was not classified as a victim of IPV.

The validity indices for the sample as a whole and by age group are shown in Table 4. Among women aged 18–24 years, the sensitivity, or probability of correctly classifying a victim of IPV through the questionnaire, was 62.5% (95% CI: 44.5–77.6). The specificity, or probability of correctly classifying a non-IPV woman, was 99.6% (95% CI: 97.0–99.9). The questionnaire presented a PPV of 95.2% (95% CI: 71.7–99.4) and a NPV of 95.1% (95% CI: 91.6–97.2). There was 147.5 times higher probability (POR) of presenting a positive result in the questionnaire among women who suffered from IPV than among those who did not suffer violence. The overall efficiency of the test, or probability of correctly classifying a woman as a victim of IPV or not, was 95.1% (95% CI: 92.6–97.7).

**Table 4:** Validity indices of the questionnaire on harassment and control by electronic tools in the detection of offline IPV

	All			18–24 years			25–29 years		
	%	95% CI		%	95% CI		%	95% CI	
**Positive cases**	5.9	4.1	8.4	7.8	5.2	11.7	3.3	1.6	6.9
**Sensitivity**	47.1	33.7	60.8	62.5	44.5	77.6	21.1	7.9	45.5
**Specificity**	99.1	97.5	99.6	99.6	97.0	99.9	98.4	95.2	99.5
**Positive Predictive Value**	85.7	67.1	94.6	95.2	71.7	99.4	57.1	20.8	87.1
**Negative Predictive Value**	94.0	91.4	95.8	95.1	91.6	97.2	92.6	88.0	95.5
**Positive odds ratio**	50.12			147.50			13.33		
**Negative odds ratio**	0.53			0.38			0.80		
**Overall efficiency of the test**	93.5	91.1	96.7	95.1	92.6	97.7	91.4	87.6	95.2

Among women aged 25–29 years, poor validity indices were observed. The sensitivity and specificity were 21.1% (95% CI: 7.9–45.5) and 98.4% (95% CI: 95.2–99.5), respectively. A PPV of 57.1% (95% CI: 20.8–87.1) and a NPV of 92.6% (95% CI: 88.0–95.5) were observed. The POR was 13.3, and the overall efficiency of the test was 91.4% (95% CI: 87.6–95.2).

## Discussion

This study explores the validity of a two-question questionnaire, HCE-2, which surveys the perception of harassment and control by electronic tools, as a screening tool for the detection of cases of IPV in young women. In the analysis, good validity indices were obtained, and these indices were better in the group of women aged 18–24 years, in which an overall test efficiency of 95.1% and a PPV of 95.2% were observed.

Cyberbullying within relationships has been described as a type of digital practice in which the aggressor exercises domination over the victim through harassing strategies that affect her privacy and intimacy [11]. This work addresses cyberbullying from a gender perspective. Most of the studies reviewed analyze cyberbullying in the context of violence in general, aimed at the detecting both victims and perpetrators of both genders. This fact make it difficult to compare our results with others.

The use of electronic tools to perpetrate different acts of violence is relatively new. Whether electronic media incite violence or are a mere new avenue for it has been discussed in previous publications, but there are still discrepancies. Electronic media have certain characteristics that differentiate them from other forms of violence: their immediacy, their lack of geographical boundaries, and the possibility of communicating online [7]. Electronic media are also heterogeneously distributed, with the young population using them the most [5]. INE (National Statistics Institute) data from 2012 (the latest before this study) indicate that 95.3% of young women are frequent users of electronic media [16].

Discrepancies are also observed in terms of terminology and classification. Some authors consider harassment and control by electronic tools an independent type of IPV, while others consider it a subtype of psychological violence [17]. Although there is no agreement on this matter, there appears to be an association between harassment and control by electronic tools in the couple and IPV towards women, called offline IPV. A study conducted in the United States in university students between 18 and 25 years of age observed a significant correlation between cases of cyberbullying within the couple and psychological, physical, and sexual violence [6]. Specifically, 95% of the participants who suffered psychological violence from their partner also suffered it by electronic tools, and conversely, those who suffered harassment by electronic tools were 28 times as likely to report psychological violence from a partner as those who did not suffer harassment. These figures support the results obtained in our study, where 95.2% of women aged 18 to 24 years who suffered cyberbullying also met the criteria of IPV. In other studies, our results are in line with others [8, 18–20] showing a strong association between harassment and control by electronic tools and psychological partner violence.

Violence by electronic media could hinder self-perception of violence, since among young people, control and harassment are often confused with attention and caring. This perception is fostered by the idea of romantic love [5], and in turn, the normalization of these behaviors favors the onset of violence [18]. However, other studies have shown that the negative consequences of IPV are present even when women are not perceived as victims of IPV [21].

The questions developed for our study have obtained good validity indices for the detection of IPV, especially in the age group of 18–24 years. Regarding scoring for the two questions, the response “rarely” was initially considered a positive score, but after observing a decrease in the overall efficiency of the questionnaire, it was given the same scoring as “never”. Recently, the short version of the Woman Abuse Screening Tool (WAST) has been validated as an IPV screening tool, in which, despite obtaining very good results, the need for improvement in detection in young populations is expressed [10]. The screening test validated in this study presents better validity indices (overall test efficiency, specificity, PPV, and POR) than WAST when applied to women aged 18 to 29 years. On the other hand, this test presents slightly lower sensitivity and NPV, although both values are considerably better in the group of younger women (18–24 years). The brevity of this test gives it an advantage over other tests geared towards young people [4, 18].

Among the limitations of this study is the nonresponse bias because it could be expected that the prevalence of IPV among women who did not want to participate in the interview would differ from the prevalence in the women who did respond. The data was collected in 2014 and the rapid change in the use of social media may be considered when interpreting these results. There may also be selection bias, as we included only women with an individual health card, though this would be minimal, since it is issued to almost 100% of the population. Added to these limitations is that we took as the gold standard a definition based on self-reported information, instead of a specialized clinical interview, and we collected information only on the most recent partner of each woman in the case that there was more than one partner in the year before the interview. The biggest strengths of the test are its brevity and simplicity, which facilitate its rapid implementation and positive acceptance by women (response rate of 100% in our sample). Since 2017, the two questions analyzed have been included in the Noncommunicable Disease Risk-Factor Surveillance System that annually monitors behavior in young people (SIVFRENT-J) in the Community of Madrid.

## Conclusions

Due to the need to improve the detection of IPV in young women and the good results of this exploratory study, it is recommended to consider cyberbullying to screen for other forms of violence in women between 18 and 24 years of age. It will be necessary to carry out more studies to ensure that the results obtained here are reliable.

## Data Availability

The General Directorate of Public Health of the Community of Madrid Health Service is the owner of this data base. They conducted the survey with the objective of epidemiological surveillance and research. The research group is part of the organization and did not need additional permission to access and use this data for research purposes. External researchers can request access to the data through ‘Portal de Transparencia’ based on the Ley 10/2019 de Transparencia y de Participación of the Community of Madrid through the following link. https://www.comunidad.madrid/transparencia/derecho-acceso-informacion-publica.

## Abbreviations

IPV: Intimate partner violence
HCE-2: Harassment and control by electronic tools questionnaire
PPV: Positive predictive value
NPV: Negative predictive value
POR: Positive odds ratio
NOR: Negative odds ratio
